# An Overview of Diagnosis and Management Strategies for Long QT Syndrome

**DOI:** 10.19102/icrm.2017.080605

**Published:** 2017-06-15

**Authors:** Susan P. Etheridge, Mitchell I. Cohen

**Affiliations:** ^1^University of Utah and Primary Children’s Medical Center, Salt Lake City, UT; ^2^University of Arizona College of Medicine and Phoenix Children’s Hospital, Phoenix, AZ

**Keywords:** Channelopathies, long QT syndrome, sudden death

## Abstract

Significant clinical, research, genetic, and therapeutic advances in the diagnosis and management of long QT syndrome (LQTS) have made the treatment of this channelopathy one of the most exciting and enlightening bench-to-bed success stories in the field of cardiology. Cascade screening identifies affected family members, and pre-symptomatic therapy saves lives. Here, we present a case of LQTS in a child and a review of the diagnostic and treatment strategies that have been introduced to date in the modern era.

## Case presentation

**Figure 1:**
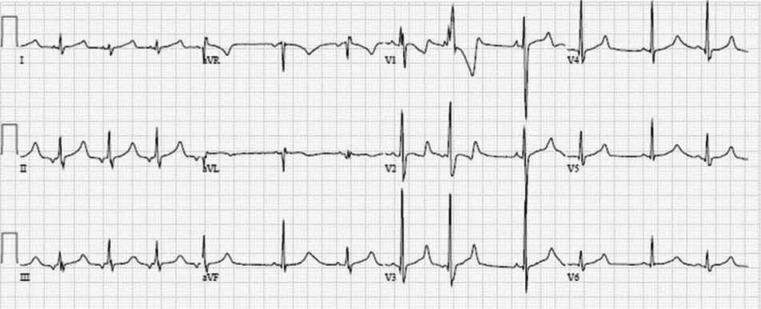
Initial 12-lead ECG in case patient with mutation R1623Q in *SCN5A* demonstrating atrial (non-sinus rhythm), ectopy, and QTc prolongation.

**Figure 2:**
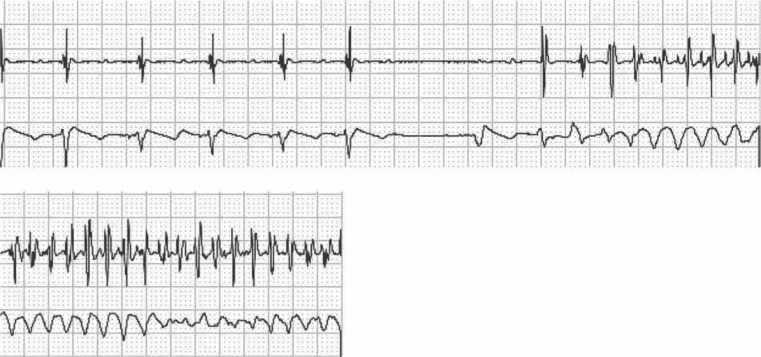
A continuous recording from an ICD interrogation from the case patient with mutation R1623Q in *SCN5A* demonstrating an event of TdP after a pause.

A five-year-old girl presented to the clinic after an episode of cardiac arrest at home. Her father heard moaning one night and went into her room to check on her. Her eyes were open but she was unresponsive and incontinent. She was gray, apneic, and pulseless, and her father began cardiopulmonary resuscitation (CPR). The paramedics were called, and she was intubated at the scene, after which she spontaneously developed respiratory effort. She was transported to the emergency department at a children’s hospital and admitted to the intensive care unit there. Her electroencephalogram, computed tomography scan, magnetic resonance image, and lumbar puncture were normal. She was started on phenytoin for a presumed seizure disorder and discharged. She had a second event six months later while walking, during which she became limp, fell, and was incontinent and not breathing. CPR was initiated again during this second episode. By the time emergency medical services arrived, she was breathing spontaneously. She was transported to the children’s hospital, where an electrocardiogram (ECG) was performed **(Figure 1)**. She was diagnosed with long QT syndrome (LQTS) and treated with nadolol and implantation of an implantable cardioverter-defibrillator (ICD). She experienced multiple appropriate ICD shocks with a single-chamber system in place **(Figure 2)**. Genetic testing was positive for mutation R1623Q in SCN5A. She was subsequently changed to a dual-chamber ICD and atrial pacing was provided; she was started on mexiletine in addition to the nadolol. No further shocks were noted.

### Disease background

Significant clinical, research, genetic, and therapeutic advances in the diagnosis and management of LQTS have made this channelopathy one of the most exciting and enlightening bench-to-bed conditions to treat in the field of cardiology. LQTS has taught us that single gene mutations can cause life-threatening arrhythmias and sudden death,^1^ that cascade screening can be used to identify affected family members, and that proper application of pre-symptomatic therapy can save lives. Importantly, LQTS, possibly more so than any other entity, has proven that knowledge gained in the basic science laboratory can quickly inform clinical practice, and that the clinical experience gained can direct and improve research. Research in LQTS has advanced our understanding of the importance of genetics in diseases that affect the heart. We have come to understand that LQTS, like many cardiac diseases of genetic origin, is characterized by incomplete penetrance and variable expressivity and, while genetic information can guide and empower therapy, misuse of genetic testing can result in misunderstanding and even harm.

LQTS is a disease of cardiac repolarization characterized by a prolonged QT interval on the ECG, a risk for syncope, seizures, and sudden death. The clinical events are a consequence of the ventricular arrhythmia, torsades de pointes (TdP), that can terminate spontaneously, resulting in syncope, or degenerate into ventricular fibrillation and sudden death. QT prolongation, a consequence of disordered cardiac repolarization, is due to mutations that encode cardiac ion channels or their accessory subunits.^2^ To date, there are 17 LQTS-susceptible genes that account for approximately 75% to 80% of clinical disease. The majority of patients are affected by the earliest of these identified: mutations in *KCNQ1* (long QT syndrome 1; LQT1),^3^ *KCNH2* (long QT syndrome 2; LQT2),^4,5^ SCN5A (long QT syndrome 3; LQT3).^6^ Loss-of-function mutations in *KCNQ1-* encoded Kv7.1 channels and KCNH2-encoded Kv11.1 channels lead to a decrease in the slowly activating potassium channel (*I*_Ks_) and rapidly activating potassium channel (*I*_Kr_), respectively. Normally, because of its fast inactivation (voltage-dependent closing), Nav1.5 does not conduct current (or does so only minimally) during the repolarization phases of the action potential. However, LQT3-linked mutations in *SCN5A* are gain-of-function mutations that impair the inactivation of Nav1.5, resulting in a late (sustained or persistent) depolarizing Na^+^ current (late sodium current, *I*_Na,L_).^2^

Genetic testing in LQTS has allowed for the development of a better understanding of the phenotype-genotype correlations.

There are clinical features that are mutation-specific but also there are features that are common to the entire group. Thus, understanding the unique features of the specific mutations can and has led to gene-specific counseling and therapy. However, it is clear that the phenotype is affected by more than the single gene mutations. Genotype–phenotype studies have shown differences in the effects of the autonomic nervous system on the pheno-type.^2^ The onset of TdP differs among LQTS genotypes. In LQT1, TdP usually occurs at fast heart rates, while in LQT2 it is often preceded by a pause,^7^ and the R-R interval immediately before TdP is significantly longer in LQT2 than in LQT1 patients. Additionally, enhanced QT interval shortening at faster heart rates was observed in LQT3 patients than other types of LQTS or normal individuals.^8^ Finally, assessing large populations has uncovered differences between individuals with identical mutations, some of which are based on autonomic nervous system responses. For example, in a large LQT1 kindred with an A341V mutation in *KCNQ1,* those with slower heart rates were at lower risk of developing symptoms.^12^

**Figure 3:**
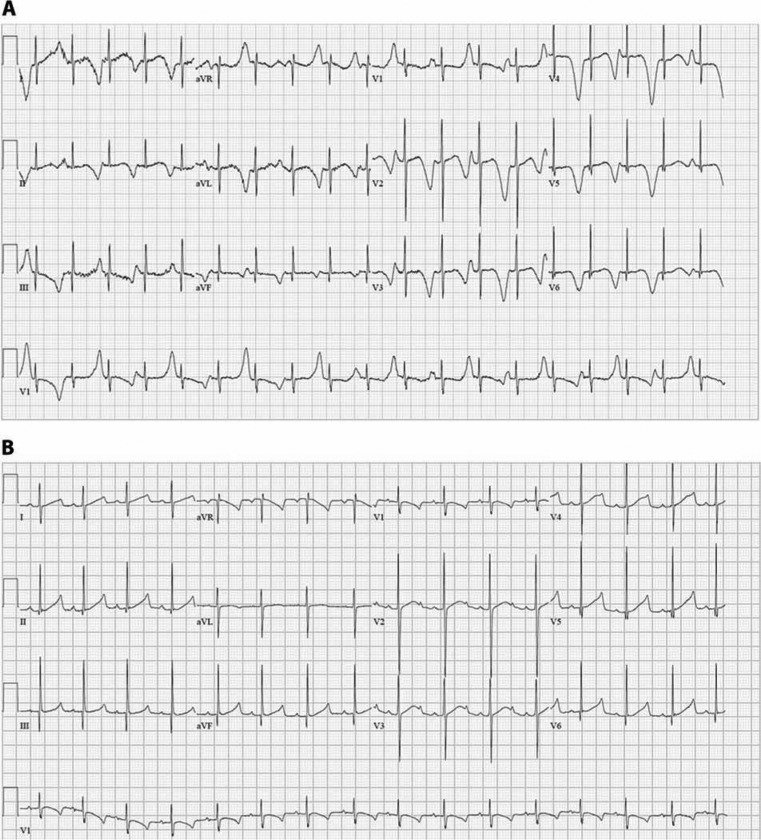
**A:** Profound QTc prolongation and T-wave alternans in a LQT1 patient with T322M mutation and a fever. **B:** Continued QTc prolongation (QTc 500 ms) but resolution of the T-wave alternans in the LQT1 patient with T322M mutation when afebrile.

In addition to the phenotypic differences noted in LQTS patients, even those carrying the same mutation, there are variations in disease expression in single individuals over time. Fever, medications, and electrolyte levels factor into disease expression. We have seen fever lengthen the QT interval and promote the appearance of T-wave alternans in LQT1 with T322M mutation **(Figure 3)**. The first description of repolarization consequences of fever was in LQT2 where repeated episodes of fever-induced TdP occurred in two related LQT2 patients (father and son) with the A558P mutation in *KCNH2*.^10^ The A558P mutant proteins were further characterized as having a dominant-negative effect on intracellular trafficking of normal Kv11.1 proteins and in reducing the temperature-dependent increases in normal Kv11.1 current, further upsetting the balance between depolarizing and repolarizing currents in favor of depolarization.^2^

Extracellular K^+^ concentration affects the QT duration in healthy people and is likely to do so to an even greater extent in those in whom mutations have resulted in compromised ion channel function. Hypokalemia is an independent risk factor contributing to reduced survival of cardiac patients and increased incidence of arrhythmic death. Although one would expect a smaller outward K^+^ current in the setting of a higher serum K^+^, I_Kr_ magnitude is paradoxically increased by an increase in extracellular K^+^. Raising the serum K^+^ level in a highly affected LQT2 population was shown to shorten the QT interval when given intravenously.^13^

### Influencing factors

Medications have long been associated with arrhythmic events in LQTS patients, unmasking the disease in some and contributing to sudden death in others. Most drugs affect KCNH2-encoded Kv11.1 channels, possibly as a consequence of the channel structure.^14^ This effect has been utilized for benefit when one considers that this is the mechanism of effect for most antiarrhythmic agents but has been a clear detriment in others, some of which have been removed from the market as a consequence of this effect.

Patient age and sex modify the QT interval and disease expression. Our youngest patients, those who are identified in utero, often have the most profound disease expression.^11^ The severe arrhythmia phenotypes noted in the fetal state can be explained in part by mutations with severe biophysical phenotypes. For example, studies of *SCN5A* mutation R1623Q, noted in sporadic LQTS cases with severe perinatal arrhythmia, identified a novel LQTS mechanism characterized by early channel reopenings and increased probability of long openings.^12^ Symptomatic infants with LQTS represent a high-risk group. Using data from the International LQTS Registry, Spazzolini et al. focused on a group with cardiac events occurring in the first year of life and on the prognostic significance of these events to age 10.^15^ They found that LQTS infants with QTc prolongation, slow heart rate, and female sex are at an increased risk for cardiac events during the first year of life. Infants who experience an episode of cardiac arrest in the first year of life were at very high risk for near-fatal or fatal cardiac events during the 10 years thereafter. Male LQTS patients experience 90% of their first cardiac events before adolescence, while female patients more often experience their first events in the post-adolescence period.^16^ This has been attributed in part to a complex interplay between sex hormones and cardiac ion channel currents. The net effect of sex hormones on the expression and function of cardiac ion channels is thought to be a lower repolarization reserve in women, rendering them more prone to QTc prolongation and TdP occurrence in the presence of a LQTS-causing mutation.^2^

***LQT1 (KCNQI) mutations.*** Themost common type of LQTS and the first to be described is LQT1. LQT1 patients are particularly vulnerable to life-threatening events during exertion, and are most responsive to β-blockers. Swimming is a known trigger, and a careful and cautious dialog in a shared risk model should discuss appropriate restrictions on physical activity.^17,18^ Our current genotype-specific management strategies in 2017 have evolved over the last decade, and will most certainly continue to do so in the future. There is information that regardless of the *KCNQ1* phenotype, that 15% of patients who have missense mutations in the transmembrane C-loop domain have a survival benefit from β-blockers.^19^ It is known that LQT1 patients with a *KCNQ1* missense mutation with a greater degree of loss of function have a greater risk of LQT1-triggered events than LQT1 patients with a C-terminal mutation. While LQT1 patients with syncope, even those using adequate β-blocker therapy, may be considered suitable candidates for ICD implantation, it may be reasonable to discuss left cardiac sympathetic denervation (LCSD) instead. ICD implantation in children is a large step, as the use of such devices will likely leave them dependent on them for the rest of their life.

***LQT2 (KCNH2) mutations.*** LQT2patients struggle in the postpartum period, especially when exposed to loud noises.

LQT2 is known to be life-threatening in females of child-bearing age when compared with male counterparts of the same age. This becomes important when counseling different-sex adolescent siblings of LQTS patients where certain recommendations given (with respect to birth control use and postpartum health maintenance) may be different. Mutations in the HERG gene encoding the rapid delayed rectifier K^+^ current I_Kr_ account for a significant proportion of LQTS. The magnitude of I_Kr_ is increased by extracelluar

K^+^. Etheridge et al. showed that using a combination of oral potassium and spironolactone to increase serum K^+^ from 4.0 ± 0.3 mEq/l to 5.2 ± 0.3 mEq/l resulted in a decrease in the QT interval from 526 ± 94 to 423 ± 36 ms.^20^ However, it is not known if this can be translated into a decreased incidence of syncope or life-threatening arrhythmias. Furthermore, children generally have normal renal function, and maintaining a sustainable elevated serum K^+^ can be challenging. Extrapolating this early observation to every single LQT2 patient and the entire potential polygenetic and molecular confounding variable would be premature.^21,22^

***LQT3 (SCN5A) mutations.*** LQT3 events occur during times of relative bradycardia and thus may manifest during sleep. Because this is a Na^+^ channel gain-of-function, Na^+^ channel-blocking agents are used. Flecainide was an early consideration; however, because of its I_Kr_ blocking effect and an unmasking of a Brugada ECG pattern in some, its use has largely gone out of favor. In some LQT3 patients with phenotypic prolonged QT intervals, shortening of the QT intervals with lidocaine or mexiletine has established a paradigm shift from β-blockers alone to consideration of the use of a Na^+^ blocker in conjunction with a β-blocker. Because LQT3 patients tend to have life-threatening events during sleep or relative instances of bradycardia, playing sports is generally allowed for these individuals. Ranolazine, a late Na^+^ channel-blocking agent, has been discussed as a potential drug for LQT3 patients,^23^ but its efficacy will likely be gene-specific.

***Neonatal LQTS.*** Symptomatic LQTS in the first year of life is concerning. Reports of extreme bradycardia as well as 2:1 functional atrioventricular (AV) block have raised awareness of LQTS in nurseries. Although early mortality rates for neonates with LQTS and 2:1 AV block have been reported to be as high as 50% to 60%,^24,25^ recent publications have shown a more optimistic outcome with the administration of β-blockers, occasional mexelitine use, and pacing.^26^ Important in this retrospective review was the observation that 75% of patients have improvement in conduction over the first year of life. Caution should be exercised before implantation of an ICD in LQTS babies with 2:1 AV block, especially in the absence of TdP or T-wave alterans. In our limited experience of neonates with 2:1 AV block and LQTS with brief runs of TdP, we have utilized a combination of a β-blocker, mexiletine, and, occasionally, a pacemaker. Future prospective studies are warranted in this rare and often severe LQTS phenotype.

### LQTS treatment

Traditionally, the management options with LQTS were primarily determined by a combination of factors including the baseline QT interval and clinical symptoms. More recently, the specific genetic abnormality has shifted from the hypothetical to early practical genotype-specific therapy. Untreated patients with LQTS are at a high risk of developing syncope and sudden cardiac death. Given that effective treatments are available in 2017, there is little excuse not to recommend treatment for symptomatic LQTS patients and in asymptomatic patients who are genotype/phenotype positive. However, there is evolving information about therapy for genotype-positive, phenotype-negative patients that could change this strategy in the near future.

Treatment for LQTS can be divided into those treated with antiadrenergic agents (β-blockers and LCSD), with an ICD and with contemporary genotype-specific therapeutic approaches. In addition, the avoidance of excessive stimulant intake, heat exhaustion, electrolyte perturbations and medications known to prolong the QT interval^1^ should be utilized in all patients.

***β-blockers.*** In one of the original landmark papers on LQTS, β-blockers and left stellate ganglionectomy significantly reduced the incidence of life-threatening cardiac events (from 53% to 9%) compared with untreated patients.^1^ In a follow-up study conducted 15 years later involving 869 patients on β-blockers, a reduction in the cardiac event rate in probands (0.97 ±1.42 to 0.31 ±0.86 events per year) and of family members (0.26 ± 0.84 to 0.15 ± 0.69) over a five-year period was shown. β-blockers remain the mainstay of treatment for LQTS.^27^ Propranolol and nadolol are the two most commonly used medications in LQTS patients. β-blockers are effective in LQT1 where the perturbation of the IKs channel makes patients sensitive to catecholamines. Vincent showed that in a large series of LQT1 patients followed for over a decade, β-blockers reduced life-threatening cardiac events by 97%.^28^ The study also highlighted the importance of non-compliance and event risk, especially in the adolescent cohort. Over time it has become apparent that not all β-blockers are equally effective in LQTS. In a study by Chockalingham involving 382 LQT1/LQT2 patients, propranolol resulted in superior QTc shortening in patients with a baseline prolonged QT interval compared with the use of meto-prolol and nadolol.^29^ Furthermore, nadolol and propranolol were equally effective in symptomatic LQTS patients, but patients on metoprolol had a greater number of breakthrough events. It was strongly recommended that metoprolol use should be avoided in LQTS. Part of the anti-arrhythmic benefits of propranolol and nadolol may be from their Na^+^ channel-blocking properties. Propranolol has been shown to have a greater effect in blocking the late non-inactivating Na^+^ current than on peak Na^+^ current, an effect not observed with metoprolol.^30^ In contrast, nadolol has an approximately 20% non-use-dependent blocking effect on the peak Na^+^ current, but not on the late current. Metoprolol has no Na^+^ channel-blocking properties.^31^ One of the more frequently discussed LQTS conundrums is the role and efficacy of β-blockers in LQT3. LQT3 patients tend to have cardiac events during states of relative bradycardia. Importantly, Wilde et al., in a recent large multicenter study of 403 LQT3 patients, found that β-blockers were associated with an 83% reduction in cardiac events in females.^32^ There were fewer events in the male patients and thus, conclusive risk reduction could not be proved, but β-blockers were noted not to be proarrhythmic.

***LCSD.*** In high-risk patients in whom β-blockers are either not effective or not tolerated, or are used in patients who are non-compliant, there should be a strong consideration for LCSD.^33^ In fact, it is our opinion that in patients, especially those with LQT1, who have syncope despite β-blockers, a LCSD procedure should be considered and discussed before the knee-jerk reaction to place an ICD. The surgical techniques (open or thoracoscopic) for performing an LCSD have been previously well described.^34–36^ In large series of patients with ongoing symptoms—most often syncope despite the use of β-blockers—LCSD have been shown to reduce the incidence of ICD shock storms (specifically, a 95% reduction in shocks), and decrease the mean QTc by 39 ms. Preliminary discussions in many journal commentaries have also noted an improvement in the QTc following LCSD in high-risk patients having previous ICD shocks.^3^

***ICD.*** Despite the well-adjudicated clinical outcomes of LQTS patients on β-blocker therapy and following an LCSD, a subset of patients remain at high risk, and thus warrant ICD implantation. Reviews discussing the various techniques to implant an ICD in children, including those requiring epicardial and pericardial systems, have been previously published.^3^ In LQTS patients who have experienced a documented cardiac arrest, the consensus at present is to implant an ICD,^33^ regardless of compliance with β-blocker therapy. This will likely evolve in the untreated LQT1 patient without profound QTc prolongation to the point where a trial of β-blockers may be considered before ICD implantation. The decision to place an ICD in a LQTS patient without an episode of prior cardiac arrest is more controversial. Our approach has been to consider an ICD in patients with continued worrisome and non-vasovagal syncope, despite the use of β-blocker therapy ± LCSD. It is important to remember that children are active and are at risk for precipitating injury to the ICD, either through somatic growth or repetitive movement. While ICDs can be life-saving, the risk of inappropriate ICD shocks remains high.^38,39^ Some inappropriate shocks can be explained by lead design flaws, though this does not explain all pediatric inappropriate shocks.^39,40^ Sinus tachycardia with inappropriate heart rate detection, short detection time, and T-wave oversensing can result in inappropriate shocks.^39–41^ ICD shocks can be psychologically debilitating and can increase the risk of depression and suicide, regardless of whether the shocks are appropriate as in ICD storms, or inappropriate. Programming efforts to minimize shocks should be sought using more lenient settings with a higher heart rate required to meet detection, and longer detection time periods provided before a shock is delivered, allowing for spontaneous termination of the arrhythmia that is common in this population.^39^

***Genotype-specific therapies.*** Over the last decade, there has been an explosion of genetic information obtained in LQTS patients. Understanding the LQTS subtype, location of the mutation, presence or absence of a haplotype deficiency, and new information regarding whole-exome sequencing have altered the genetic landscape and inundated clinical electrophysiologists with a wealth of information. Filtering off the “noise” to understand the true mutations can be challenging, and requires the involvement of individuals who are actively engaged in the contemporary clinical management of LQTS.^42^ Even if one were to siphon off the “noise” and false variants of unknown significance, genotype-specific LQTS management is still in its embryonic state. We have taken the liberty of providing some considerations regarding genotype-specific LQTS therapies in 2017. Without a doubt, this will appear completely different within a few years. However, before embarking on any therapeutic approach to LQTS, there should be a discussion and shared decision-making between the physician, the patient, and the patient’s parents or guardians.^43^ No two LQTS patients are the same, even if they carry the same genetic mutation. Environmental factors, sex, genetic modifiers, and polymorphisms all contribute to the phenotype. In addition, prior familial experience (such as perception from parents who have lost a child with LQTS) have emotional effects that can shape a treatment course. Careful discussions regarding the pros and cons of all LQTS treatments need to be discussed. These discussions should be open and rational, and should occur multiple times over the child’s progression into adolescence and young adulthood. Shared decision-making is reasonable when all parties are informed about the risks and benefits, and understand the variety of treatment options available.^43^

The patient in the illustrative case was managed in the early 2000s, and her management today would be different. Nowadays, we have an improved understanding of the overlap between seizures and LQTS, and we would have come to the correct diagnosis more quickly, hopefully before the second cardiac arrest. Genetic testing is undertaken early in the course of patient management, as it is clear that knowledge about present genetic mutations affects prognosis and therapy. In the future, we may even decide that an ICD is not needed in a patient like the one in this case. However, should an ICD be used in a child with atrial arrhythmias and pause-dependent TdP, the addition of an atrial lead and rate-smoothing pacing would be advantageous. Also, the addition of a Na^+^ channel-blocking agent alone might be the best second line of therapy, even in this child with proven high-risk LQT3. Nonetheless, it is important to know that this child is now a 22-year-old woman who is stable without further ICD shocks and is on appropriate therapy with mexiletine and nadolol.

### Concerning LQTS patients

In conclusion, the following patient cohorts should be remembered as individuals who should be kept in mind when considering LQTS.

- Patients with QTc ≥ 550 ms (especially > 600 ms) on multiple resting ECGs;
- LQT1 patients with exertional syncope despite the use of β-blocker therapy;
- LQT2 female patients of child-bearing age with non-sustained ventricular arrhythmias;
- patients with multiple mutations in a single gene (ie, multiple *KCNQ1* mutations);
- patients experiencing recurrent non-vasovagal syncope despite the use of β-blocker therapy;
- LQT3 patients with extreme bradycardia or long pauses;
- symptomatic neonates with significant QT prolongation and/or arrhythmias;
- neonates with LQT functional 2:1 AV block, bradycardia, and torsades or T-wave alternans;
- adolescents with a history of noncompliance and/or high-risk behaviors; and
- patients with Timothy syndrome (LQT8).
